# Case Report: Next-generation metagenomic sequencing in the diagnosis of *Brucella*-associated joint infections—a case series analysis and comprehensive literature review

**DOI:** 10.3389/fmed.2025.1688037

**Published:** 2025-11-13

**Authors:** Jing Duan, Xianzeng Li, Yanhui Hu, Feng Pang, Yinguang Cao, Zhiqing You

**Affiliations:** 1 Department of Clinical Laboratory, Liaocheng People's Hospital, Liaocheng, Shandong, China; 2 Department of Clinical Laboratory, Shenxian Central Hospital, Liaocheng, Shandong, China; 3 Department of Joint Surgery, Beijing Jishuitang Hospital Liaocheng Hospital, Liaocheng, Shandong, China

**Keywords:** metagenomic next-generation sequencing, brucellosis, joint infection, diagnosis, literature review

## Abstract

**Background:**

The application of next-generation metagenomic sequencing (mNGS) in the diagnosis of human brucellosis, particularly in cases of joint brucellosis infection, remains under-explored, with rarely no case reports available in the literature. We present the first case series focusing on the application of mNGS in the diagnosis of *Brucella* joint infections. The results indicate that mNGS plays a crucial role in diagnosing *Brucella* joint infections, serving as a valuable complement, particularly for culture-negative patients.

**Case presentation:**

This study presents a comprehensive analysis of four cases of human joint brucellosis diagnosed using mNGS on the BGI sequencing platform, involving three male and one female patients aged from 42 to 63 years, all of whom had documented epidemiological exposure histories. mNGS successfully identified *Brucella* sequences in all cases, with additional diagnostic findings including a positive *Brucella* agglutination test in Patient 1, positive joint fluid cultures in Patients 3 and 4, and no positive results in Patient 2. Following surgery and targeted antibiotic therapy, all patients exhibited clinical improvement and favorable follow-up outcomes.

**Conclusion:**

These findings underscore the utility of mNGS as a critical diagnostic tool for joint brucellosis infections and highlight its potential as a complementary approach in cases of culture-negative joint infections. In cases where clinical suspicion of joint infection persists despite the absence of identifiable etiological evidence, the implementation of mNGS is strongly advised to facilitate timely and accurate clinical decision-making.

## Introduction

Brucellosis is a zoonotic infectious disease caused by *Brucella* (primarily *Brucella melitensis*, *Brucella abortus* and *Brucella suis*), which is widely prevalent globally and poses a significant public health threat, especially in regions with developed animal husbandry. Conservative estimates indicate approximately 2.1 million new cases annually worldwide, with particularly severe impacts in Africa and Asia (1, 2). The disease manifests not only through prolonged fever, fatigue, and multi-system dysfunction in patients but also exerts significant socio-economic consequences due to workforce depletion and substantial healthcare expenditures. Among the diverse complications associated with *Brucella* infection, osteoarticular manifestations are particularly prevalent, occurring in approximately 2–77% of cases, with spondylitis and infections of the hip and knee joints representing the most frequently observed presentations. Affected individuals commonly exhibit clinical features such as joint swelling, pain, restricted range of motion, and systemic manifestations including undulant fever and profuse sweating (3, 4). Conventional etiological diagnostic approaches, including serological assays and bacterial culture techniques, demonstrate limited sensitivity and necessitate extended processing durations, yielding pathogen detection rates below 30% in cases of joint infections. This diagnostic inefficiency contributes to therapeutic delays, potentiates the risk of articular tissue destruction, and consequently elevates both patient disability rates and healthcare expenditures (5). The utilization of mNGS technology in pathogen identification has garnered increasing attention in recent years, owing to its high-throughput capacity, rapid turnaround time, enhanced accuracy, and ability to simultaneously detect multiple pathogens within clinical specimens, thereby providing robust support for diagnostic decision-making. This study represents the first systematic retrospective analysis of four cases of joint *Brucella* infection diagnosed with mNGS assistance, demonstrating that mNGS serves as a critical diagnostic tool for joint *Brucella* infection and offers a valuable supplementary approach for culture-negative cases.

## Case reports

### Case 1

**Figure 1:**
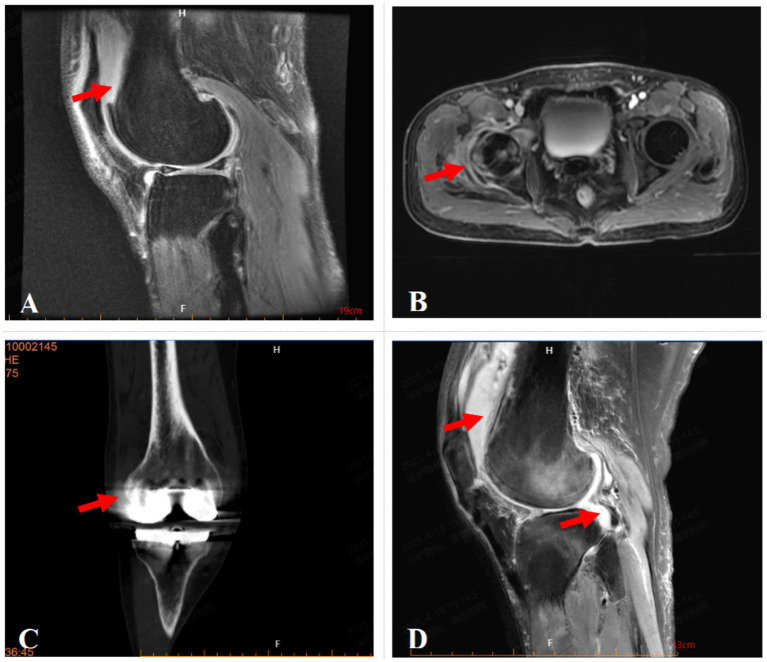
Imaging of the affected joints in four patients. **(A)** Fat-suppressed MRI image of case 1 shows anterolateral subcutaneous edema and suprapatellar bursal effusion. **(B)** Contrast-enhanced MRI image of case 2 demonstrates abnormal signal intensity with restricted diffusion and enhancement in the periaritcular soft tissues of the right hip, suggestive of inflammation with abscess formation. **(C)** CT image of case 3 shows Knee Prosthesis and swelling of the periarticular soft tissues. **(D)** Fat-suppressed MRI image of case 4 shows joint effusion and suprapatellar bursal fluid with periarticular soft tissue edema.

A 55-year-old male farmer presented with a history of right knee pain and weakness persisting for three years, exacerbated by physical exertion. The symptoms were significantly aggravated following an accidental right knee sprain two weeks prior to admission. On April 26, 2022, the patient was hospitalized with a diagnosis of severe osteoarthritis of the right knee joint. Clinical examination revealed a normal body temperature (37 °C), knee joint swelling, localized skin temperature elevation, and a 5° varus deformity. Tenderness was noted on the anteromedial aspect of the right knee, with restricted flexion-extension range (5°–90°) and a positive patellar ballottement test. Magnetic resonance imaging (MRI) demonstrated injuries to the medial and lateral menisci, patellar ligament, anterior and posterior cruciate ligaments, and medial and lateral collateral ligaments. Additionally, osteochondral damage and bone marrow edema were observed in the medial femoral and tibial condyles, accompanied by subcutaneous edema on the anterolateral knee and effusion in the joint cavity and suprapatellar bursa (Figure 1A). Laboratory findings shows elevated white blood cell count (WBC) and C-reactive protein (CRP) level, along with increased erythrocyte sedimentation rate (ESR), indicate significant inflammation, possibly suggestive of septic arthritis (Table 1). While joint fluid culture (bioMérieux blood culture system) was negative, mNGS identified *Brucella* (14 reads), and the *Brucella* agglutination test yielded a positive result. Following confirmation of *Brucella* joint infection, the patient was transferred to Jinan Infectious Disease Hospital for subsequent treatment. The six-month follow-up showed that the patient has recovered well.

**Table 1:** Summary of patient characteristics, clinical and laboratory findings.

Indexs	Case1	Case2	Case3	Case4
Gender/Age	M/55	M/42	M/63	F/47
Affected joints	Right knee	Right hip	Right knee	Right knee
Medical history	severe osteoarthritis	No significant past medical history	Total knee arthroplasty hypertension	hypertension, diabetes mellitus, and hyperlipidemia
Clinical symptoms	Aggravation of knee pain	Hip tenderness and limited mobility	Recurrent right knee pain accompanied by swelling and tenderness	Intermittent knee pain
Imaging findings	Meniscal and multiligamentous knee injury	Bone marrow edema involving the right hip with bilateral joint effusions	Joint effusion with surrounding soft tissue swelling	severe osteoarthritis, featuring meniscal tears, multiligamentous injury, synovitis, and joint effusion.
Treatment regiments	DOX (0.1 g bid) + RIF (0.9 g qd)	DOX (0.1 g bid) + RIF (0.6 g qd) + CRO (2.0 g IV bid)	DOX (0.1 g bid) + RIF (0.6 g qd) + CRO (2.0 g IV bid)	DOX (0.1 g bid) + RIF (0.9 g qd) + CRO (2.0 g IV bid)
Time from onset to diagnosis	16 days	48 days	185 days	14 days
Outcomes	Relief	Relief	Relief	Relief
Follow-ups	6 months no recurrence	6 months no recurrence	6 months no recurrence	1 months no recurrence
Route of transmission	NA	Exposure to cattle and sheep	herd sheep	Risks from unpasteurized milk, cross-contamination
Temp on admission (°C)	37.0	36.6	36.8	36.5
WBC (×10^9^/L)	10.25*	12.01*	7.64	5.36
Synovial WBC (×10^9^/L)	7.80*	5.56*	5.76*	16.16*
CRP (mg/L)	105.14*	47.73*	45*	40.08*
ESR (mm/h)	67*	73*	45*	57*
ABR	Positive	Negative	Negative	NA
SFC	Negative	Negative	Positive	Positive
mNGS reads	Brucella sp. (14/−)	Brucella sp. (5,021/−)	B.melitensis (3,010/3)	B.melitensis (17,415/26)

ABR, agglutination test for brucellosis; SFC, synovial fluid culture; CRO, cefatriaxone; DOX, doxycycline; RIF, rifampicin; NA, Not available.

*, represents this index is above the normal reference range.

### Case 2

A 42-year-old male farmer presented with a 37-day history of right hip injury sustained during heavy lifting, accompanied by progressive limitation of right hip joint mobility. The patient was admitted on December 4, 2023, due to worsening hip pain. Initial physical examination revealed normal body temperature (36.6 °C), tenderness upon percussion of the right greater trochanter, positive tenderness at the midpoint of the right inguinal region, restricted right hip joint mobility, a positive right “4” sign, and intact sensation and muscle strength in both lower limbs with normal dorsalis pedis artery pulsation. MRI demonstrated bone marrow edema in the right femoral head and acetabulum, soft tissue edema surrounding the right hip joint, and bilateral hip joint effusion (Figure 1B). Laboratory investigations revealed elevated inflammatory markers, including WBC, CRP and ESR, indicate significant inflammation which possibly caused by infection (Table 1). Empirical antibiotic therapy with moxifloxacin was initiated. Subsequent diagnostic evaluations, including joint fluid and blood cultures, yielded negative results, and the Brucella agglutination test was also negative. However, mNGS of the joint fluid identified Brucella species (reads: 5,021). Considering the patient’s occupational exposure to cattle and sheep, a definitive diagnosis of Brucella joint infection was established. The patient underwent arthroscopic hip debridement, followed by a 3-month course of doxycycline (100 mg twice daily) and rifampin (600 mg once daily), supplemented with ceftriaxone (2.0 g IV every 12 h) for 1 month. Clinical improvement was observed, with resolution of fever, significant alleviation of joint pain, and absence of other discomforts. Follow-up assessments at 1, 3, and 6 months post-discharge confirmed sustained clinical stability.

### Case 3

A 63-year-old male farmer presented with a two-year history of right knee joint pain of unknown etiology, which exacerbated following physical activity and necessitated a total knee arthroplasty (Figure 1C). Approximately 18 months postoperatively, the patient developed recurrent right knee pain accompanied by swelling and tenderness, prompting hospital admission on January 30, 2024. The patient’s medical history included hypertension. Clinical examination upon admission revealed a normal body temperature (36.8 °C), with restricted flexion and extension of the right knee joint (range of motion: 5° extension to 90° flexion) and a positive patellar ballottement test. Conservative management proved ineffective, necessitating revision surgery of the knee prosthesis. Laboratory investigations such as WBC, CRP and ESR indicated signs of inflammation and joint infection cannot be excluded (Table 1). Empirical antibiotic therapy was initiated, and synovial fluid culture (blood culture bottles, bioMérieux) remained negative after 5 days of incubation. Subsequent mNGS identified Brucella (3,010 reads) and *Brucella melitensis* (3 reads), and synovial fluid culture yielding positive results after a prolonged incubation time of 72 h. Epidemiological Investigation revealed prior sheep farming activities 2 years earlier, confirming a diagnosis of brucellosis-associated joint infection. The patient was treated with a combination of oral doxycycline (100 mg twice daily) and rifampin (600 mg once daily) for 3 months, supplemented with ceftriaxone (2.0 g IV every 12 h) for 1 month. Clinical improvement was observed, with resolution of right knee pain, swelling, and tenderness, and absence of fever or systemic symptoms. Follow-up evaluations at 1, 3, and 6 months demonstrated sustained clinical remission.

### Case 4

A 47-year-old female agricultural worker presented with a six-month history of persistent dull pain and weakness in the right knee joint, exacerbated by physical activity, without identifiable precipitating factors. Initial conservative management failed to alleviate symptoms, and MRI revealed a meniscal injury. On September 6, 2024, the patient underwent partial meniscectomy with joint debridement. Postoperatively, she continued to experience intermittent knee pain, which was refractory to conservative interventions, including oral analgesics and intra-articular sodium hyaluronate injections. She was subsequently readmitted on February 10, 2025, with a diagnosis of right knee osteoarthritis (Figure 1D). The patient’s medical history included hypertension, diabetes mellitus, and hyperlipidemia. Clinical examination upon admission revealed a normal body temperature (36.5 °C), mild soft tissue swelling around the right knee, negative patellar ballottement, slightly elevated local skin temperature, approximately 10° varus deformity, positive tenderness, and restricted flexion-extension range of motion (25–45°). On February 12, 2025, the patient underwent arthroscopic exploration and debridement of the right knee joint with drainage tube placement, followed by 3-day anti-infective therapy with vancomycin (1 g IV drip q12h). However, laboratory findings includes high level of WBC, CRP, ESR, and elevated synovial fluid white blood cell count (16,160 × 106/L), suggestive of significant inflammation (Table 1). Synovial fluid mNGS identified Brucella (reads: 17,415) and *Brucella melitensis* (reads: 26), with only the second culture yielding positive results. The patient’s history of consuming unpasteurized milk and inadequate separation of raw and cooked food preparation surfaces supported the epidemiological diagnosis. The patient was treated with rifampicin 0.9 g PO daily for 3 months, doxycycline 0.1 g PO daily for 3 months and ceftriaxone 2 g IV daily for 1 month, resulting in significant alleviation of joint pain and absence of fever or other notable symptoms. One-month follow-up post-discharge confirmed the patient’s stable condition.

## Discussion and conclusion

This case series reports four cases of *Brucella* joint infection from Shandong, China, all diagnosed with the assistance of mNGS. The patients, all farmers, ranged in age from 43 to 63 years, with the right knee or hip joints primarily affected. The main clinical symptoms included swelling, pain, fatigue, and limited mobility in the affected joints, with symptoms worsening after exertion or activity. Most patients experienced prolonged joint symptoms, with other symptoms being atypical. Laboratory tests showed elevated inflammatory markers such as blood WBC, CRP, and ESR, while imaging revealed joint effusion and periarticular soft tissue edema indicative of inflammation. Distinguishing *brucella* arthritis requires focusing on key clinical patterns. Unlike the insidious, destructive nature of tuberculous arthritis, it often presents acutely with significant fever and night sweats. In contrast to the symmetrical polyarthritis of rheumatoid arthritis, brucellosis typically affects a single large joint. It is clearly differentiated from the non-inflammatory, mechanical pain of osteoarthritis by its systemic symptoms and inflammatory signs. Definitive diagnosis relies on specific tests like culture, serology or mNGS to confirm *Brucella* infection.

The laboratory diagnosis of *Brucella* infection currently employs three primary methodologies: serological antibody detection, polymerase chain reaction (PCR), and bacterial culture. While serological assays offer rapid results, their diagnostic utility is compromised by cross-reactivity, leading to both false-positive and false-negative outcomes. Key limitations of serological testing include the lack of standardized interpretative criteria, which exhibit variability across laboratories and are influenced by clinical and epidemiological factors. Furthermore, these assays often demonstrate sub-optimal specificity due to inter-species cross-reactivity and reduced sensitivity during the early stages of infection. Although PCR-based methods exhibit relatively high sensitivity and specificity, their diagnostic accuracy is contingent on prior clinical assumptions and the expertise of the clinician. Bacterial culture, regarded as the diagnostic gold standard, is constrained by a low positivity rate and prolonged turnaround time. Additionally, its diagnostic yield is significantly diminished by the empirical use of antibiotics, rendering it unsuitable for meeting the demands of rapid clinical decision-making (6, 7). Recent advancements in mNGS have highlighted its exceptional potential in pathogen identification, particularly in complex clinical scenarios such as bloodstream infections, central nervous system infections, and joint infections. This technology offers distinct advantages, including the ability to detect pathogens without prior knowledge of their identity and its comprehensive coverage of nucleic acid sequences. Compared to conventional diagnostic methods, mNGS has demonstrated superior performance, especially in identifying rare or fastidious pathogens. A review of preliminary studies on the diagnostic efficacy of mNGS for peripheral joint infections reveals that the sensitivity of this method ranges from 73.83 to 100%, specificity from 86.3 to 95.2%, positive predictive value (PPV) from 88 to 97.9%, and negative predictive value (NPV) from 75.44 to 100%. These findings underscore the robustness of mNGS as a diagnostic tool for joint infections (Table 2). Furthermore, the accuracy and sensitivity of mNGS in diagnosing joint infections following empirical antibiotic treatment are significantly higher than those of culture. This is primarily because the use of antibiotics can affect the growth of pathogenic microorganisms, thereby significantly reducing the positive rate. In contrast, mNGS directly sequences the nucleic acids of pathogenic microorganisms in joint fluid, bypassing the cultivation step. The viability of pathogens has minimal impact on mNGS, resulting in a better positive rate (8).

**Table 2:** The sensitivity, specificity, PPV, and NPV of mNGS in diagnosing joint infections.

Researcher/Time	Cases	Sequencing Platform	Sensitivity (%, 95 CI%)	Specificity (%, 95 CI%)	PPV (%, 95 CI%)	NPV (%, 95 CI%)
2023 (14)	107	Illumina Nextseq 550 DX	73.83 (NA)	91.49 (NA)	90.80 (NA)	75.44 (NA)
2023 (15)	91	BGISEQ-500	91.3 (82.0–96.7)	86.3 (65.0–97.0)	95.4 (87.2–99.0)	76.0 (54.8–90.6)
2021 (16)	35	BGISEQ-50	93 (NA)	90 (NA)	88 (NA)	95 (NA)
2020 (17)	63	BGISEQ-500	95.6 (83.6–99.2)	94.4 (70.6–99.7)	97.7 (86.5–99.9)	89.5 (65.5–98.2)
2020 (18)	85	BGISEQ-500	95.9 (86.3–98.9)	95.2 (77.3–99.2)	97.9 (89.1–99.6)	90.9 (72.2–97.5)
2019 (19)	37	BGISEQ-500	100 (NA)	92.31 (NA)	96 (NA)	100 (NA)

PPV, Positive Predictive Value; NPV, Negative Predictive Value; NA, Not available.

To date, a limited but growing number of cases have demonstrated the utility of mNGS in diagnosing brucellosis, particularly in clinically challenging scenarios. Based on the reviewed literature, mNGS has successfully identified *Brucella* species in at least 21 reported cases from various regions, including both endemic and non-endemic areas, with a notable concentration of reports from China. The central nervous system (CNS) is the most frequently reported site of infection, involved in the vast majority of cases (19/21), highlighting the critical role of mNGS in diagnosing neurobrucellosis. Other affected sites include the spine and the eyes, as evidenced by single case reports of spondylitis and infectious endophthalmitis. mNGS has proven valuable in confirming the diagnosis, especially when traditional methods like serology or culture were negative, delayed, or inconclusive. The technique reliably detected *Brucella* (predominantly *B.melitensis*) reads directly from clinical samples such as cerebrospinal fluid (CSF), with reported reads ranging from very low (1 species-specific reads) to very high (13,762 reads), demonstrating high sensitivity. Furthermore, mNGS has been instrumental in identifying the pathogen in cases with atypical presentations or without clear epidemiological history, facilitating timely and targeted antimicrobial therapy, which commonly includes combinations of doxycycline, rifampicin, and third-generation cephalosporins (Table 3). Apart from case reports, retrospective studies likewise demonstrates mNGS has a diagnostic sensitivity surpassing 85% for both *Brucella* bacteremia and central nervous system infections, while concurrently reducing the time to diagnosis to less than 48 h (9, 10). However, the application of mNGS in the diagnosis of *Brucella*-associated joint infections remains inadequately explored, with a notable paucity of systematic case studies in the existing literature. To our knowledge, only one comprehensive study has evaluated mNGS for diagnosing *Brucella* joint infections, revealing superior sensitivity (83.33%) and accuracy (86.20%) versus conventional methods (serum agglutination test: 62.50, 63.79%; culture: 20.83, 34.49%) (11). The remaining three studies did not specifically delineate the diagnostic efficacy of mNGS for *Brucella*-associated joint infections; however, their findings collectively demonstrated that *Brucella* represents a prevalent microbial pathogen in joint infections, and mNGS technology exhibits robust capability in its accurate identification (8, 12, 13).

**Table 3:** Summary of preliminary literature on mNGS diagnosis of brucellosis.

Ref/publish time	Cases	Gender/Age	Affected sites	Initial symptoms	mNGS reads (genus/species)	Other etiological evidences	Area/contury	Epidemiological history	Treatment regimens
2021 (20)	1	M/39	CNS	Obnubilation and coma	*B.melitensis* (199/4)	PCR/serum agglutination test	Shanghai China	YES	CRO (2.0 g IV bid) + DOX (0.1 g tid) + RIF (0.15 g tid)
2023 (21)	1	M/67	Spine/L 4–7	Chest and back pain	*B.melitensis* (−/20)	RBPT	Zhejiang China	YES	DOX (0.1 g bid) + RIF (0.45 g qd)
2025 (22)	1	F/10	CNS	Headache, fever, vomiting, altered consciousness, seizures, urinary incontinence	*B.melitensis* (−/32)	*Brucella* antibody testing+cultrue	Yunnan China	NA	CRO (0.1 g/kg IV q24h, 1mo) + DOX (2.2 mg/kg q12h) + RIF (15 mg/kg q24h, 6mo)
2025 (23)	1	F/41	CNS	Fever and headaches	*Brucella abortus* (−/2)	RBPT	Guangdong China	YES	DOX (0.1 g bid) + RIF (0.6 g qd), 6Mo + CRO (2gbid 6w)
2025 (24)	3	F/3.5	CNS	Dizziness, vomiting, drowsiness	B.melitensis (−/162)	RBPT	Inner Mongolia China	YES	SMZ-TMP, RIF (4Mo)
		F/14		Headache, vomiting, lethargy	B.melitensis (−/5,252)	RBPT		YES	CRO iv + DOX + RIF (4Mo) 🠆SMZ-TMP + RIF + DOX (2Mo)
		M/14		Headache, vomiting and lethargy	B.melitensis (−/59)	RBPT+blood culture		YES	CRO iv + DOX + RIF + SMZ-TMP (4Mo)🠆SMZ-TMP + RIF + DOX (2Mo)
2025 (25)	1	F/37	CNS	Dizziness and numbness and weakness of the limbs	*B.melitensis* (165/2)	RBPT+tube agglutination test	Inner Mongolia China	NA	DOX (0.1 g bid) + RIF (0.6 g qd) + Pred (0.03gqd) + SMZ-TMP (2Tab qd); 6Mo
2023 (26)	1	M/32	CNS	Fever, headache, dizziness, and memory complaints	*Brucella* sp. (486/−)	Serum agglutination test	Northeast Brazil	NA	CRO + RIF + DOX;6Mo
2025 (27)	1	F/35	CNS	Headache, nausea, non-spraying vomiting, fearless fever	*Brucella* sp. (289/−)	No other positive evidences	Ganshu China	NA	DOX (0.1 g bid) + RIF (0.15 g qd) + Pred (0.02 g qd)
2024 (9)	4	M/60	CNS	Fever, chills and cough	*B.melitensis* (−/4)	Blood culture	Zhejiang China	YES	Omadacycline (0.3 g qd) + LEV (0.5 g qd)
		F/60		Fever, lumbago	*B.melitensis* (−/1,314)	Culture +RBPT		YES	DOX (0.1 g bid) + RIF (0.45 g qd)
		F/71		A painless and enclosed mass at back	*B.melitensis* (−/30)	Pus culture		NO	DOX (0.1 g bid) + RIF (0.45 g qd) + MXF (0.4 g qd)
		M/55		Lumbago, numbness in the lower limbs and fever	*B.melitensis* (−/58)	RBPT		YES	DOX (0.1 g bid) + RIF (0.6 g qd)
2020 (28)	1	M/6	CNS	Fever, enervated, irritable, sleepy, photophobia	*B.melitensis* (−/1)	RBPT+Blood culture	Shaanxi China	YES	RIF + SMZ-TMP; 2Mo
2022 (29)	1	F/57	Eye	Fever with red eyes and eyelid edema	*B.melitensis* (−/13,762)	No other positive evidences	Jiangsu China	YES	DOX (0.01 g bid) +RIF (0.9 g qd)
2018 (30)	4	M/38	CNS	Fever, headache, hearing loss, back pain	*Brucella* sp. (30/−)	PCR + CSF culture	Beijing China	NA	NA
		F/46		Hearing loss, weight loss	*Brucella* sp. (11/−)	PCR + RBPT		NA	NA
		F/38		Headache, hearing loss	*Brucella* sp. (24/−)	PCR + RBPT		NA	NA
		M/49		Fever, seizures	*Brucella* sp. (104/−)	PCR + RBPT		NA	NA
2017 (31)	1	F/11	CNS	Fever, headache, nausea, back pain	*Brucella* sp. (277/−)	PCR and Rose Bengal tests	California America	NO	RIF + DOX

CRO, cefatriaxone; DOX, doxycycline; RIF, rifampicin; MXF, moxifloxacin; LEV, levofloxacin; SMZ-TMP, compound sulfamethoxazole; Pred, prednisone; Mo, month; W, week; Tab, tablet; IV, Intravenous Injection, RBPT, Rose Bengal plate test, *B. melitensis*, B.melitensis.

Through the analysis of four cases of *Brucella* arthritis, we found that the clinical features were consistent with those reported in the literature, primarily manifesting as involvement of weight-bearing joints. Traditional joint fluid culture is prone to negative results due to low pathogen load, early antibiotic use, or non-optimal culture conditions for fastidious microorganisms. In contrast, mNGS technology, leveraging its advantages in nucleic acid detection, can provide reports in an average of 28 h, significantly shortening the time for etiological diagnosis by approximately 2–3 days, and is less affected by antibiotics. The sequence reads (14–17,415) were consistent with similar studies, confirming its sensitivity and reliability. All patients had a history of contact with cattle, sheep, or their products, indicating that epidemiological investigation is crucial for the diagnosis of *Brucella* infection. In terms of treatment, all four patients were treated with the standard regiment of doxycycline combined with rifampin, supplemented with short-term intravenous use of ceftriaxone. Even though some cases initially received empirical treatment, timely adjustment based on mNGS results did not significantly affect the efficacy. This study is the first to report a series of cases of *Brucella* joint infection successfully diagnosed through mNGS technology, systematically analyzing their clinical characteristics, laboratory test indicators, and complementarity with traditional methods. Our study demonstrates that mNGS can serve as a powerful tool for diagnosing *Brucella* joint infections, particularly in patients with prior antibiotic exposure and negative cultures, where it often provides robust etiological evidence.

This case analysis offers crucial insights for clinicians. When encountering unexplained joint inflammation, especially with symptoms like fever and sweating, a detailed history of contact with livestock or unpasteurized dairy products is key to suspecting *Brucella* infection, which is critical to avoid misdiagnosis in non-endemic areas. In such scenarios where traditional cultures are negative or time is limited, mNGS serves as a rapid and sensitive alternative, significantly shortening the diagnostic timeline and enabling early targeted treatment. Moreover, after diagnosis, treatment must adhere to guidelines with standard regimens, such as doxycycline combined with rifampin, and ensure a sufficient course to eradicate intracellular pathogens and prevent relapse. There are also several notable limitations. First of all, the small sample size and absence of a control group preclude the establishment of causal relationships and hinder the ability to account for potential confounding variables and the findings may be influenced by selection bias and publication bias, thereby limiting the representativeness and generalizability of the results. Furthermore, the retrospective nature of the study also introduces the possibility of incomplete or non-standardized data collection. Thirdly, as a semi-quantitative technique, the read count in mNGS cannot directly reflect bacterial viability or disease severity, nor can it provide crucial treatment guidance information such as antibiotic susceptibility. And the high testing costs and complex data analysis limit its widespread adoption in primary hospitals. Lastly, mNGS data was used for species-level identification but was not sufficient for robust whole-genome sequencing and single-nucleotide polymorphism (SNP) analysis to explore the phylogenetic relationships between our isolates and global strains which would be valuable in tracing transmission networks.

## Data Availability

The datasets presented in this study can be found in online repositories. The names of the repository/repositories and accession number(s) can be found below: https://bigd.big.ac.cn/gsa/browse/CRA030534, CRA030534.

## Glossary

mNGS: metagenomics next generation sequencing
WBC: white blood cell count
CRP: C-reactive protein
ESR: erythrocyte sedimentation rate
MRI: magnetic resonance imaging
PCRq: polymerase chain reaction
PPV: Positive Predictive Value
NPV: Negative Predictive Value
NA: Not available
ABR: agglutination test for brucellosis
SFC: synovial fluid culture
CRO: cefatriaxone
DOX: doxycycline
RIF: rifampicin
MXF: moxifloxacin
LEV: levofloxacin
SMZ-TMP: compound sulfamethoxazole
Pred: prednisone
Mo: month
W: week
Tab: tablet
IV: Intravenous Injection
RBPT: Rose Bengal plate test
*B. melitensis*: *B.melitensis*
